# 

**Published:** 1827-12

**Authors:** 


### MONTHLY LIST OF MEDICAL BOOKS.

[*No Books can be entered on this List except a copy be sent for the purpose; the titles of works having frequently been transmitted to us, as published, which have not appeared for weeks, or even months, after.*]

A Treatise on the Diseases of the Chest and on Mediate Auscultation, by R. T. H. Laennec, m.d. Regius Professor of Medicine in the College of France, Clinical Professor to the Faculty of Medicine of Paris, Physician to Her Royal Highness the Duchess of Berri, &c. &c. &c. Second Edition, greatly enlarged. Translated from the French, with Notes, and a Sketch of the Author's Life, by John Forbes, m.d. Member of the Royal College of Physicians, and senior Physician to the Chichester Infirmary. With Plates. —8vo. pp. 722. London, 1827.

An Introduction to the Comparative Anatomy of Animals; compiled with constant Reference to Physiology, and elucidated by twenty Copper-plates; by C. G. Carus, Med. et Phil. Doct. &c. Translated from the German, by R. T. Gore, Member of the Royal College of Surgeons in London. In two Volumes. Vol. I.—8vo. pp. 371. London, 1827.

A Letter addressed to the Faculty of Physic in London, on the illegal and unwholesome Bye-laws of the College of Physicians of London, establishing a Monopoly in favor of the Graduates of Oxford and Cambridge. By Henry Robertson, m.d. of the University of Edinburgh.—8vo. London, 1827.

A Treatise on the Cutaneous Diseases incidental to Childhood: comprehending their Origin, Nature, Treatment, and Prevention. By Walter C. Dendy, Surgeon to the Royal Infirmary for Children, &c. &c.—8vo. pp. 289. London, 1827.

An Introductory Lecture, delivered at the Medical Theatre, Dean-street, Borough, on the 1st of October, 1827, wherein are explained the Nature, Objects, and Advantages of Anatomy, not only to Medical Science, but to Art and Science in general. By Francis Snaith, Surgeon, and Demonstrator of Anatomy.—8vo. pp. 40. London, 1827.

A short Account of the Small-Pox which prevailed at Bridport and its Neighbourhood, in the Summer of 1826. With Remarks on Vaccination, and the degree of Protection it was found to afford during the Prevalence of the Epidemic. By John Strang, Surgeon, r.n.—8vo. pp. 53. Bridport, 1826.

A Manual of Midwifery, for the use of young Practitioners of both Sexes. By WM. MACLURE, Surgeon, &c.—18mo. pp. 34. London, 1827.

Medical Botany, No. XI.—containing Atropa Belladonna (a new engraving, the former not having been botanically correct), Humulus Lupulus, Sinapis nigra et alba, Amygdalus Communis, and Capsicum Annuum.

*** The plates continue to be executed in the best style.

Flora Medica, No. I.: containing Botanical Descriptions, Natural History, Chemical Properties and Analysis, Medical Properties and Uses, &c. &c. &c.—This Number contains Amygdalus Communis, Sinapis Alba, Solanum Dulcamara, Papaver Rhœas, Daphne Mezereum, and Malva Sylvestris. —The plates are done on stone, and astonishingly well for the price (2s. 6d.) There is also a copious and useful letter-press.

### METEOROLOGICAL JOURNAL,

### NOTICES.

*The Papers of* Sir G. GIBBES *and* Dr. BARON *have been unavoidably postponed: they shall appear next month.*

*The Communications of* Mr. LYONS *and* Mr. HAMILTON *have likewise come to hand.*

*Several* ANNOUNCEMENTS *of Works in the Press have been received; the necessity for the insertion of which is partly superseded by the Titles of all new Books sent for Review being included in the* MONTHLY LIST OF BOOKS. *The Proprietors beg to suggest, that such Notices may be given on the Wrapper as* ADVERTISEMENTS, *for which a* Scale of Prices *is on the back of the Title-page.*

### INDEX

TO THE

THIRD VOLUME OF THE NEW SERIES

OF THE

### London Medical and Physical Journal.

PAGE

Abscess of the chest cured by an operation . . . 372

—— liver bursting into the pericardium . . . 451

——, remarkable case of . . . 178

Absorbents, Fohmann on (analysed) . . . 337

Accouchement after the death of the mother . . . 468

Action against the Lancet, notice concerning . . . 475

Amputation at the Hip-joint, case of . . . 228

Anchylosis of the Hip cured by an operation . . . 138

Aneurisms of the Aorta, 94, 174, 478

—— Heart . . . 552

Ankle, injury of, from gymnastic exercises . . . 305

Animal Mechanics, analysis of . 542

Antimony in Inflammation . 464

Aorta, rupture of, without aneurism . . . 15, 19

Apothecaries, their new regulations . . 378, 474, 565

Arachnitis, case of, treated with Quina and Opium . . . 265

Arsenic used in the cure of Ague in a child six weeks old . 364

Arteries, on diseases and wounds of . . . . 25

Arteria Iliaca Communis, ligature applied to . . . 371

—— externa tied . 97

Atmospheric pressure, observations on its influence on the circulation . . . 125

Books, monthly lists of, 92, 187, 283, 380, 475, 571

Bite of a snake, case of fatal 358

Bladder, perforation of the . 317

Borax, its action on the Uterus 468

PAGE

Brain, pathology of, cases illustrating . . . . 498
Bronchotomy, case of, in Laryngitis . . . . 366
—— ditto —— . 370

Cancer of the Tongue, cases of . 274
Cantharides, preservation of . 556
Cæsarean section, two cases of 402
Carotid artery tied for a fleshy tumor . . . 408
Catalepsy, case of . . 392
Circulation, Dr. Carson on the effect of atmospheric pressure on the . . . 125
——, Report on Dr. Barry's Memoir on the . 492
Children, Mr. Haden on (notice of) . . . . 351
Chorea, pathology of . . 457
Coccus Cacti, natural history of . . . . 278
Coecum, ruptured, case of . 169
College of Physicians, remarks on the . . . . 373
—— versus Dr. Harrison . . . 280
—— Surgeons, notice of the petition against the . . 90
Common Iliac tied by Dr. Mott 371
Constipation, cases of 264, 487
Convulsions, Puerperal, case of 102
Copper, Sulphate of, in Diarrhœa 461
Correspondence between Dr. Harrison and Dr. Chambers 85
—— between the Censors and Dr. Harrison . 375
Cow-pox, on constitutional inaptitude to . . . 381
——, remarks upon the preceding . . . 568
Cupping-glass, new form of . 279
Cutaneous Diseases, Mr. Dendy on (analysed) . . 532

PAGE

Death under suspicious circumstances . . . 484
Diseases of Females, Dr. Hall on (analysed) . . . 51
Disinfecting Liquid of Soda, remarks on the . . 82, 83
Dissection, case of wound received in . . . 171
Dublin Hospital Reports, vol. iv. notice of . . . 162

Ear, experiments on the functions of the . . . 546
——, bad effects of stimulating applications to the . . 279
Engravings, Mr. Mayo's, of the Brain, notice of . . 63
Epilepsy, case of . . 505
Erysipelas epidemic in Jamaica 553
————, on the use of the Lunar Caustic in . . . 223
Excision of carious Joints, cases of 179
Experiments on Tendons . 550
Extra-uterine Fœtation, case of 314
Eye, Guthrie on the Operative Surgery of (analysed) . 245

Fascination of Snakes . . 549
Fees, extracts concerning, from Mr. Wadd's Maxims and Mems. 470
Females, Dr. Dewees on the Diseases of (analysed) . . 326
Femoral Artery, unusual variety of the . . . . 352
Fever, Dr. Gregory's case of . 512
——— Dr. Bow on . . 360
——— Dr. Hewett's remarks on 104
Fistula Lachrymalis, cases of . 477
Food, means of administering to insane persons . . 324
——, comparative degrees of nutrition of different kinds of . 278
Fracture, ununited, of the Humerus cured by pressure . 44
Fungus Hæmatodes of the Eye, case of . . . 502

Gums, Phagedenic Ulceration of, in children . . . 363
Gymnastic exercises, observations on . . . 504

Heart, Ossification of the . 480
——— presentation, case of . 561
Hernia, cases of, 20, 411, 419, 420
Herpes, application of diluted Prussic Acid to . . 468
Homœopathia, or Art of curing Diseases founded on Resemblances, (account of) . 78

PAGE

Hospital, St. George's, report on Mr. Sleigh's charges against . 183
Hydrocephalus, Dr. Chapman on 30
Hydrocyanic Acid, detection of, in those poisoned with it . 276
Hydrophobia, case of, in a surgeon 457
Humerus, fractures of . . 10
Hunterian Oration, Mr. Thomas's (analysed) . . . 71

Infibulation, case of . . 558
Inflammation of the Veins . 455
————————, on the propagation of . . . . 265
———————— of the Tongue cured by incisions . . 267
Inquest extraordinary . . 564
Intermittents treated with large doses of Arsenic . . 403
——————, bleeding in the cold stage of . . 1, 75
Intestinal Calculus, case of . 505
Iron, piece of, in the thorax for fourteen years . . 453
——, Muriate of, in Softening of the Stomach . . . 463

Jaw, amputation of portions of the . . . . 468
Joints, carious, cases of excision of . . . . 179

Kidney, suppuration of the . 486
King's Evil, history of its cure by the royal touch . . 561

Labia, on sudden swelling of the 421

Lepra cured by Fumigations . 364

Life, remarks on . . 405

List of new Fellows and Licentiates . . . . 571

Lists of Books, 92, 187, 283, 380, 475, 571

Lunatics, pauper, powers of the commissioners over . . 569

Lungs, test of being sound . 554

——— Peripneumonic Abscess of the . . . 139

Lusus Naturæ, case of . 50

Luxation, ill effects of reducing when of long standing . 557

Malaria, observations on . 555

Mechanics, Animal, in the Library of Useful Knowledge, (analysed) . . . 542

Medicine, Clinical, analysis of Dr. Bischoff on . . 168

Medico-Chirurgical Transactions (notice of) . . . 450

PAGE
Medico-Chirurgical Society, their first meeting . . . 472
——— Botanical Society, their meetings . . . 474
Meteorological Journals, 92, 188, 284, 380, 476, 572
Midwifery, notice of a pamphlet on the Impropriety of Men being employed in the Practice of 73
Morphia, extraction of, from dry Poppy-heads . . 469
Mortification of the extremities, case of . . . 514
Moxa, application of, in cases of Stiff-joint . . . 309
———, Mr. Wallace on (analysed) 344

Nævus Maternus, singular case of 48
Nerves, structure of the . 451
Neuralgia, case of, cured with Iron . . . . . 361
Notices to Correspondents, 92, 188, 284, 380, 476, 572
Nutrition of the Fœtus, observations on . . . 221

Obituary of Mr. Shaw . . 187
Opium, method of detecting minute quantities of . . 277
———, case where an infant recovered, by keeping up artificial respiration, after taking thirty drops of the tincture of 80
Ophthalmia, purulent, use of Chloride of Lime in . . 386
Ossification of the Heart, case of 480

Penis, strangulation of, from the socket of a candlestick . 557

Pericarditis, case of . . 508

Perineum, Calculus in the . 467

———, laceration of . 101

Phrenitis, case of, treated with Croton-oil . . . 47

Physics, Elements of, by Dr. Arnott (analysed) . . 231

Physicians, College of, their dispute with Dr. Harrison . 373

Placenta, detachment of the, by injecting the veins . . 559

Poison of various Serpents, experiments on the . . 355

Poisoning, supposed case of . 322

——— from the bite of a Snake, case of . . . 358

Pomatum-pot found in the Vagina 557

Principles of Medical Science, by Dr. Shute (analysis of) . 151

Pulsation of Veins . . 361

Pulse, Dr. Rucco on (analysis of) 538

PAGE
Pus, signs by which its presence may be detected . . 175

Quina, quantity of, consumed . 277
——— Sulphate of, its adulteration . . . . ib.

Regulations of the Apothecaries' Company . . . 378
Répertoire géneral d'Anatomie, No. IV. (analysed) . . 68
Report of prevalent Diseases, 85, 182, 279, 373, 472, 565
——— Medical Cases, by Dr. Bright (analysis of) . . 518
Respiration, on the power of suspending, possessed by the Amphibia and Birds . . 451
Resuscitation of still-born Children . . . . 99
Rheine, a peculiar substance in Rhubarb . . . 276
Rheumatism, Dr. Scudamore on (analysis of) . . . 427
Rhododendron Chrysanthemum, notice of . . . 379
Roses, method of increasing the odour of . . . 556
Rupture, receipt for making a 275

Scrotum, removal of, for calculous degeneration . . 516
Scurvy, cases of . . 482
Secale Cornutum, use of the 469, 210, 214

Serpents, experiments on the poison of various . . 355
———, fascination of . 549
Skin, method of applying medicines by the . 168, 464
———, its supposed power of absorbing, remarks on . . 131
Skull, fractures of the . 204, 207
Sloughing Sores, at the Lock Hospital . . . . 285
Small-pox after Vaccination, cases of . . . 221
Specifics, account of . . 562
Spinal Diseases, Dr. Harrison on (analysed) . . . 258
Stomach, singular, described . 353
———, softening of, Muriate of Iron in . . . 463
Stricture of the Œsophagus, case of, from swallowing Sulphuric Acid . . . . 310
Strictures of the Urethra, Mr. Stafford's method of dividing 292
Styptic, notice concerning a new . . . 91

PAGE

Suckling, bad effects of, when protracted . . . 119
Surgical Pathology of the Larynx, Mr. Porter on the (analysis of) 439
Suspicious death, case of . 484

Tail, account of a remarkable production in the human subject, resembling a . . 167
Tendons, experiments on . 550
Testicle, diseases of the . 298
Thigh, remarks on fractures of the 466
Transfusion, case of . 45, 497
Turpentine, oil of, changes which it undergoes from exposure . 277

Ulceration of the Gums in children, Charcoal in . . 363
University of London, professors of the . . . 186
Urethra, cases of Hemorrhage from the . . . 80

PAGE

Urethra, operation for the restoration of a portion of the . 373
Urine, retention of, with puncture of the bladder . . 397
———, extravasation of . 197
Uterus, anterior obliquity of, observations on . . 216
——— filled with blood . 495

Vaccination, constitutional inaptitude for . . . 381
Veins, pulsation of . 170, 361
Vision, on the focus of . . 546
Voice, remarks upon the . 168
Vomiting from pregnancy, dependent upon a morbid state of the Uterus . . 560

Westminster Medical Society, their first meeting . . 473
Windpipe, loss of a portion of the 468

---

### ORIGINAL PAPERS AND CASES.

### INJURIES OF THE HEAD.

Case of extensive Fracture of the Skull, successfully treated. By Mr. Cox 204
——— Fractured Skull. By Mr. Hunter . . . . 207

### PATHOLOGY OF THE BRAIN.

Aneurismal Tumor, between the Brain and Sella Tursica. By Dr. Chevalier . . . . . . . . . 498
Fungous Hæmatodes in the right Eye, with an anomalous Tumor at the Base of the Brain. By Martin Ware, Esq. . . . 502

### DISEASES OF THE HEART AND AORTA.

Case of Rupture of the Aorta, near its Arch. By Thomas Rose, Esq. Surgeon to *St. George's Hospital* . . . . . 15
Memoranda of two Cases of Rupture of the Aorta, without Aneurism . 19
Ossification of the Heart. By Mr. Rainy . . . . 480

### ANEURISM.

Aneurism of the Aorta which burst into the Pericardium. By Mr. Rose 94

————————, terminating in Rupture into the Pericardium. By Dr. Hewett, (*St. George's Hospital*) . . . . 99

————————, which burst into the Œsophagus . . 96

————————, bursting externally through the left Mamma . 478

### DISEASED AND WOUNDED ARTERIES.

Case of Diseased and Wounded Arteries, treated principally at *St. Thomas's Hospital*, by B. Travers, Esq. F.R.S. . . . 25

PAGE

### FEVER.

On the Use of Blood-letting in the Cold Stage of the Paroxysm of Intermittent Fever. By T. RIDGWAY, M.D. . . . . . 1

A comparative View of the various Alterations of Structure observed in the fatal Cases of continued Fever, which have occurred at *St. George's Hospital*, between July 1st, 1826, and July 1st, 1827. By Dr. HEWETT 104

Cases illustrating the Efficacy of Arsenic in Intermittent Fever. By Mr. TEEVAN . . . . . . . . . 403

### HYSTERICAL CATALEPSY.

Case of Hysterical Catalepsy; with Remarks. By JOHN NORTH, Esq. 392

### HYDROCEPHALUS.

Pathology and Treatment of Hydrocephalus. By N. CHAPMAN, M.D. 30

### HEMORRHAGE.

Case in which the external Iliac Artery was tied under peculiar circumstances, by Dr GIBBS, at the *General Naval Hospital, St. Petersburgh* 97

### PULMONARY ABSCESS.

Case of Peripneumonic Abscess of the Lungs; with some Remarks. By W. F. CHAMBERS, M.D. Physician to *St. George's Hospital* . . 189

### FRACTURE OF THE HUMERUS.

Cases of Fracture of the Neck of the Humerus, treated by Mr. TRAVERS, at *St. Thomas's Hospital.* With Remarks, by Mr. HAINWORTH . 10

### INJURY OF THE ANKLE.

Case in which fatal Consequences arose from an Injury to the Ankle, in some Gymnastic Exercises. By THOMAS ROSE, Esq. . . 305

### HERNIA.

Cases of Strangulated Hernia, operated on by Mr. H. EARLE, at *St. Bartholomew's Hospital* . . . . . . . . 20

——— Hernia, treated at *St. Bartholomew's Hospital*, with Observations. By H. EARLE, Esq. F.R.S. &c. . . . . . . 411

Case of Strangulated Femoral Hernia. By Mr. GREEN, *St. Thomas's Hospital* . . . . . . . . . 419

——— ———————. By Mr. TYRRELL, *ditto* . 420

### URINARY ORGANS.

Extravasation of Urine, treated by Mr. GREEN, at *St. Thomas's Hospital* 197

———————, treated at *St. George's Hospital*, by Mr. JEFFREYS 201

Case of Retention of Urine from Strictures, relieved by puncturing the Bladder, and followed by Hæmaturia. By W. ANDREWS, Esq. . 397

Remarks upon a method of dividing Impermeable Strictures within the Canal of the Urethra; accompanied with Cases. By Mr. STAFFORD 292

Case of Perforation of the Bladder and successive Formation of Calculi. By P. L. SERPH, Surgeon . . . . . . . 317

### DISEASES OF THE TESTICLE.

Cases illustrative of the Pathology and Surgical Treatment of some of the Diseases of the Testicles; with Observations. By Mr. BRODIE 298

### PHYSIOLOGY.

Influence of Atmospheric Pressure on the Circulation of the Blood. By Dr. CARSON . . . . . . . . . 125

On the Supposed Function of the Skin as an Absorbent. By Dr. ROBERTSON . . . . . . . . . 131

PAGE
Dr. CARSON's Opinions respecting the Influence of Atmospheric Pressure on the Circulation of the Blood, examined by Dr. N. ARNOTT . 218
Memoranda respecting some Investigation into the Manner in which the Nutrition of the Fœtus is effected, which have recently been made by C. LEE, M.D. . . . . . . . . . 221
On Life. By Sir GEORGE SMITH GIBBES . . . . 405
Report, by Dr. EDWARDS, sen. and M. JULES CLOQUET, on Dr. Barry's Memoir relative to the Circulation of the Blood . . . 492

### ERYSIPELAS.

On the Cure of Erysipelas of the Face by the external Application of the Nitrate of Silver. By JOHN HIGGINBOTTOM, Esq. . . 223
Dr. STORER on the Cure of Erysipelas . . . . . 227

### OPHTHALMIA.

On the Employment of Chloruret of Oxide of Calcium in the Treatment of Purulent Ophthalmia. By Dr. VARLEZ, Surgeon to the *Military Hospital, Brussels* . . . . . . . . 386
——— Ditto ———. By Mr. GUTHRIE, at the *Royal Infirmary for Diseases of the Eye* . . . . . . . . . 389

### SMALL-POX AND VACCINATION.

Cases and Remarks on Small-Pox after Vaccination. By the late T. CHEVALIER, Esq. F.R.S. &c. . . . . . . . 122
On Constitutional Inaptitude to Cow-Pox. By GEO. GREGORY, M.D. 381
Remarks on the above . . . . . . . . 568

### CONSTIPATION.

Constipation of the Bowels during twenty-one days, successfully treated. By J. JONES, Esq. . . . . . . . . . 487
——————————— of twelve days' standing, cured. By JAMES ALEXANDER, M.D. . . . . . . . . 489

### SCURVY.

Case of Land Scurvy, successfully treated . . . . . 482

——— Sea Scurvy, with Effusion into the Chest. Fatal . . 483

—————————, with Effusion. Fatal . . . . 484

### PATHOLOGY OF THE UTERUS.

Distention of the unimpregnated Uterus with Blood. By J. PAUL, M.D. 495

### TRANSFUSION.

Case of Transfusion. By Mr. R. P. PHILPOTT, of Brighton . . 497

### CÆSAREAN SECTION.

Memorandum of two Cases of Cæsarean Section, performed at the *Civil Hospital, Prague.* Communicated by Dr. LEFEVRE . . . 402

### MIDWIFERY.

On the Resuscitation of apparently Still-born Children. By Mr. TOOGOOD 99
Case of Laceration of the Perineum. By Mr. WILLIAMS . . 101
——— Puerperal Convulsions. By Mr. JONES . . . 102
Cases of tedious Labour, in which the Secale Cornutum was exhibited; with Remarks. By JAMES GUTHRIE, Esq. . . . . 210
Case of successful Exhibition of the Ergot of Rye in lingering Labour. By Mr. CLAY . . . . . . . . . . 214
Remarks on the Treatment to be adopted during Labour in Cases of the anterior Obliquity of the Uterus. By Mr. NORTH . . . 216

PAGE

Case in which the Bones of a Fœtus were passed through an Opening in the Abdomen, by which Aperture the Catamenia afterwards flowed. Treated at *St. George's Hospital*, by Mr. GUNNING . . . 314

Cases of Bloody Infiltrations in the Labia Pudendi. By Dr. DEWEES 421

### DISEASES OF CHILDREN.

Observations on the injurious Effects which frequently arise in Children from protracted Suckling. By E. MORTON, M.D. (*Metropolitan Infirmary for Children*) . . . . . . . . 119

### MISCELLANEOUS.

Case of Ununited Fracture of the Arm, successfully treated by Pressure at *St. George's Hospital*, by Mr. BRODIE . . . . . 44

—— Transfusion after Uterine Hemorrhage. By Mr. DOUGLAS FOX 45

—— Phrenitic Inflammation, benefited by Croton-oil. By Mr. FROST 47

—— singular Nævus Maternus. By G. BENNETT, Esq. . . . 48

—— Lusus Naturæ. Communicated by W. D. ROLFE, Esq. . . 50

On the Treatment of Anchylosis, by the Formation of Artificial Joints. By Dr. BARTON, Surgeon to the *Pennsylvania Hospital* . . 138

Successful Amputation at the Hip-joint. By VALENTINE MOTT, M.D. 228

Observations on Sloughing Sores. By G. G. BABINGTON, Esq. Surgeon to the *Lock Hospital* . . . . . . . . . 285

Cases and Observations illustrating the Application of Moxa to the Treatment of Chronic Affections of the Limbs. By J. BOYLE, Esq. (*Middlesex Infirmary*) . . . . . . . . . 309

Stricture of the Œsophagus, from swallowing Sulphuric Acid, successfully treated. By Mr. TAYLOR . . . . . . . 319

Supposed Case of Poisoning by Oxymuriate of Mercury. By Mr. FROST 322

On the Means of administering Food to Insane Persons, in Cases of Resistance. By CALEB WILLIAMS, Surgeon to the *Retreat, York* . 324

Account of a Fleshy Tumor, for which the Carotid Artery was tied. By CHARLES MAYO, Esq. Surgeon to the *Winchester Hospital* . . 408

Report of Cases treated at the *Aberdeen New Dispensary*, and communicated by ALEXANDER RAINY, senior Pupil . . . . . 477

Cases of Fistula Lachrymalis . . . . . . . ib.

Case of Suffocation, from Carelessness . . . . . . 484

—— Suppuration of the Kidney; with Dissection . . . . 486

Some Remarks on Gymnastic Exercises. By J. MACARTNEY, M.D. . 504

Case of Intestinal Calculus. Communicated by Mr. WICKHAM . 505

—— Epilepsy. Detailed by the PATIENT . . . . . ib.

—— Pericarditis, successfully treated by S. D. BROUGHTON, Esq. . 508

—— (Fever?) By Dr. GREGORY (*St. George's and St. James's Disp.*) 512

Singular Case of Mortification. By Mr. GREEN (*St. Thomas's Hospital*) 514

Removal of the whole of the Scrotum. By VALENTINE MOTT, M.D. . 516

---

### CRITICAL ANALYSES.

Dr. MARSHALL HALL on some of the more important Diseases of Females 51

Mr. MAYO'S Engravings, illustrating the Structure of the Brain, &c. in Man 63

Répertoire général d'Anatomie et de Physiologie, No. IV. . . 68

Mr. H. LEIGH THOMAS'S Hunterian Oration . . . . 71

On the Impropriety of Men being employed in the Business of Midwifery 73

Principles of Medical Science and Practice. By HARDWICKE SHUTE, M.D. 151

Dublin Hospital Reports and Communications, &c. Vol. IV. . . 162

A Treatise on Clinical Medicine, by Dr. BISCHOFF. From the German, by Dr. COPE . . . . . . . . 168

Elements of Physics, or Natural Philosophy, General and Medical, explained independently of Technical Mathematics. By Dr. N. ARNOTT 231

PAGE

Lectures on the Operative Surgery of the Eye. By Mr. GUTHRIE . 245
Spinal Diseases, illustrated with Cases and Engravings. By Dr. HARRISON 258
A Treatise on the Diseases of Females. By WM. P. DEWEES, M.D. . 326
Das Sangadersystem der Wirbelthiere. Von VINCENZ FOHMANN . 337
Pathological Inquiry respecting the Action of Moxa. By Mr. WALLACE 344
Mr. HADEN on the Management and Diseases of Children . . 351
Dr. SCUDAMORE's Treatise on the Nature and Cure of Rheumatism . 427
Mr. PORTER on the Surgical Pathology of the Larynx and Trachea, &c. 439
Medico-Chirurgical Transactions. Vol. XIII. . . . . . 450
Reports of Medical Cases. By RICHARD BRIGHT, M.D. F.R.S. &c. . 518
Mr. DENDY's Treatise on the Cutaneous Diseases incidental to Childhood 532
Introduction to the Science of the Pulse. By JULIUS RUCCO, M.D. . 538
Library of Useful Knowledge. Animal Mechanics. No. IX. . . 542

---

COLLECTANEA . . *Pages* 75, 167, 264, 352, 451, 546
INTELLIGENCE . . . 85, 182, 279, 373, 472, 565
METEOROLOGICAL REGISTER 92, 188, 284, 380, 476, 572

---

### PLATES.

Mr. BENNETT's Case of singular Nævus Maternus . *to face page* 48

Mr. ROLFE's Case of Lusus Naturæ . . . . . ib.

Dr. BARTON's Case of Anchylosis . . . . . . 138

Mr. WARE's Case of Fungous Hæmatodes in the right Eye, &c. . . 502

### NAMES OF AUTHORS,

*Of whose Works, Observations, &c. either a detailed Account, or more or less general View, is given in the present Volume.*

PAGE

Alexander, Dr. his case of Constipation removed by the use of a syringe 489

Andrews, Mr. his case of Retention of Urine . . . 397

Arnott, Dr. his observations on Dr. Carson's opinions with regard to Atmospheric Pressure . . . . . . . 218

———, his Elements of Physics, (analysed) . . . 231

Babington, Mr. on Sloughing Sores . . . . . 285

Ballingall, Dr. his remarks on Fractures of the Thigh . . 466

———, his case of Calculus in the Perineum . . 467

Barton, Dr. his case of artificial Hip-joint . . . 138

Barry, Dr. Report on his Memoir on the Circulation . . . 492

Bennet, Mr. his case of singular Nævus Maternus . . . 48

Bow, Dr. on Fever . . . . . . . . 360

Boyle, Mr. on Moxa . . . . . . . 309

Breschet, M. on Aneurism of the Heart . . . . 552

Bright, Dr. analysis of his Report of Medical Cases . . . 518

Brodie, Mr. on the Testicle . . . . . . . 298

——— his case of Ununited Fracture treated by Pressure . 44

Broughton, Mr. his case of Pericarditis . . . . 508

Carson, Dr. on the Influence of Atmospheric Pressure on the Circulation 125

Chambers, Dr. on Peripneumonic Abscess of the Lungs . . 189

Chapman, Dr. on Hydrocephalus . . . . . . 30

Chevalier, the late Mr. his cases of Small-pox after Vaccination . 122

———, Mr. his case of Aneurismal Tumor in the Brain . . 498

Clay, Mr. his exhibition of the Ergot of Rye in lingering Labour . 214

Cope, Dr. his translation of Bischoff's Clinical Medicine (analysis of) 168

Cox, Mr. his case of Fracture of the Skull . . . . 204

Dendy, Mr. on the Diseases of Children (analysed) . . . 532

Dewees, Dr. on the Diseases of Females (analysed) . . . 326

——— on sudden Swelling of the Labia Pudendi . . 421

Earle, Mr. his cases of Hernia . . . . . 20, 411

Elliotson, Dr. on Sulphate of Copper in Diarrhœa . . . 461

Fohmann, M. on the Absorbents (analysed) . . . . 337

Forbes, Dr. his case of Inflammation of the Veins . . . 455

Fox, Mr. his case of Transfusion . . . . . 45

Frost, Mr. his case of supposed Poisoning . . . . 322

——— Phrenitis treated with Croton-oil . . 47

Gibbes, Sir G. his observations on Life . . . . 405

Gibbs, Dr. case in which he tied the external Iliac Artery . . 97

Granville, Dr. on the Disinfecting Liquid of Soda . . . 82

Green, Mr. his case of Extravasation of Urine . . . 197

——— Hernia . . . . . 419

——— Mortification of the Extremities . . . 514

Gregory, Dr. on Constitutional Inaptitude to Cow-pox . . 381

———, his case of Fever . . . . . 512

Gunning, Mr. his case of Extra-uterine Fœtation . . . 314

Guthrie, Mr. on the Operative Surgery of the Eye (analysed) . 245

——— on Purulent Ophthalmia . . . . 389

———, Mr. James, on Secale Cornutum . . . . 210

Haden, Mr. notice of his work on Children . . . . 351

Hainworth, Mr. on Fracture of the Neck of the Humerus . . 10

Hall, Dr. M. on the Diseases of Females (analysed) . . 51

PAGE
HARRISON, Dr. his Correspondence with Dr. CHAMBERS . . 85
——————— the Censors of the College of Physicians . . . . . . . . 375
——— on Spinal Diseases (analysed) . . . 258
HEWETT, Dr. his cases of Aneurism of the Aorta . . . 96
——— on Fever . . . . . . . 104
HIGGINBOTTOM, Mr. on the Cure of Erysipelas by Lunar Caustic . 223
HUNTER, Mr. his case of Fracture of the Skull . . . 207
JEFFREYS, Mr. his case of Extravasation of Urine . . . 201
JONES, Mr. his case of Puerperal Convulsions . . . 102
——————— Constipation, lasting twenty-one days . . 487
LEE, Dr. R. on the Nutrition of the Fœtus . . . . 221
LYONS, Mr. his test of sound Lungs . . . . . 554
MACARTNEY, Dr. his observations on Gymnastics . . . 504
MAYO, Mr. CHARLES, case in which he tied the Carotid Artery . 408
MAYO, Mr. H. his Engravings of the Brain, (notice of) . . 63
MORTON, Dr. on Protracted Suckling . . . . . . 119
MOTT, Dr. case in which he tied the common Iliac Artery . . 371
——— his case of Amputation at the Hip-joint . . . 228
——————— removal of the Scrotum, for Calculous Degeneration 516
NORTH, Mr. his case of Catalepsy . . . . . 592
——— observations on the anterior Obliquity of the Uterus . 216
PAUL, Dr. his case of Distention of the unimpregnated Uterus with Blood 495
PORTER, Mr. on the Larynx (analysed) . . . . . 427
RAINY, Mr. A. his Report of Cases treated at the Aberdeen New Dispensary 477
RIDGWAY, Dr. on Bleeding in the Cold Stage of Intermittents . 1
ROBERTSON, Dr. on Absorption by the Skin . . . . 131
ROLPH, Mr his case of Lusus Naturæ . . . . 50
ROSE, Mr. on Injury of the Ankle . . . . . 305
———, case of Aneurism of the Aorta . . . . 94
——————— Rupture of the Aorta . . . . 15
RUCCO, Dr. on the Pulse (analysed) . . . . 538
RUMBALL, Mr. on the Focus of Vision . . . . 546
SCUDAMORE, Dr. on Rheumatism (analysed) . . . 427
SERPH, Mr. his case of Perforation of the Bladder . . . 317
SHAW, Mr. Obituary of . . . . . . . 187
SHUTE, Dr. his Principles of Medical Science (analysed) . . 151
SLEIGH, Mr. his charges against St. George's Hospital, Report on . 183
STAFFORD, Mr. his method of dividing Strictures of the Urethra . 292
STORER, Dr. on the cure of Erysipelas by Lunar Caustic . . 227
TAYLOR, Mr. his case of Stricture of the Œsophagus . . . 310
TEEVAN, Mr. on Arsenic in Ague . . . . . . 403
THOMAS, Mr. notice of his Hunterian Oration . . . 71
TOOGOOD, Mr. his cases of Resuscitation of Still-born Children . 99
TRAVERS, Mr. on Diseased and Wounded Arteries . . . 25
TYRRELL, Mr. his case of Hernia . . . . . . 420
VARLEZ, Dr. on Purulent Ophthalmia . . . . . 389
WADD, Mr. extracts from his Maxims and Memoirs . . . 470
WALLACE, Mr. on Moxa (analysed) . . . . . 344
WARE, Mr. his case of Fungous Hæmatodes of the Eye . . 502
WHEATSTONE, Mr. his Experiments on Hearing . . . 546
WILLIAMS, Mr. his case of Laceration of the Perineum . . 101
——————— on administering Food to Insane Persons . . 32[illegible]
WICKHAM, Mr. his case of Intestinal Calculus . . . 505

J. and C. Adlard, Printers, Bartholomew Close.

2100

